# Generation and Evaluation of a Genome-Scale Metabolic Network Model of *Synechococcus elongatus* PCC7942

**DOI:** 10.3390/metabo4030680

**Published:** 2014-08-20

**Authors:** Julián Triana, Arnau Montagud†, Maria Siurana, David Fuente, Arantxa Urchueguía, Daniel Gamermann, Javier Torres, Jose Tena, Pedro Fernández de Córdoba, Javier F. Urchueguía

**Affiliations:** 1 Universidad de Pinar del Río “Hermanos Saíz Montes de Oca”, Pinar del Río 20100, Cuba; E-Mail: jtriana@upr.edu.cu; 2 Instituto Universitario de Matemática Pura y Aplicada, Universitat Politècnica de València, València 46022, Spain; E-Mails: armontag@mat.upv.es (A.M.); masiupa@upv.es (M.S.); dafueher@upv.es (D.F.); pfernandez@mat.upv.es (P.F.C.); 3 Biozentrum, Universität Basel, 4056 Basel, Switzerland; E-Mail: arantxa.urchueguia@gmail.com; 4 Instituto de Física, Universidade Federal do Rio Grande do Sul (UFRGS), Porto Alegre 91501-970, Brazil; E-Mail: danielg@if.ufrgs.br; 5 Instituto Universitario de Medio Ambiente y Ciencia Marina, Universidad Católica de Valencia “San Vicente Mártir”, València 46001, Spain; E-Mails: javier.torres@ucv.es (J.To.); josetena@ucv.es (J.Te.).

**Keywords:** genome-scale metabolic network reconstruction, systems biology, metabolic pathways, flux balance analysis, biological databases

## Abstract

The reconstruction of genome-scale metabolic models and their applications represent a great advantage of systems biology. Through their use as metabolic flux simulation models, production of industrially-interesting metabolites can be predicted. Due to the growing number of studies of metabolic models driven by the increasing genomic sequencing projects, it is important to conceptualize steps of reconstruction and analysis. We have focused our work in the cyanobacterium *Synechococcus elongatus* PCC7942, for which several analyses and insights are unveiled. A comprehensive approach has been used, which can be of interest to lead the process of manual curation and genome-scale metabolic analysis. The final model, *i*Syf715 includes 851 reactions and 838 metabolites. A biomass equation, which encompasses elementary building blocks to allow cell growth, is also included. The applicability of the model is finally demonstrated by simulating autotrophic growth conditions of *Synechococcus elongatus* PCC7942.

## 1. Introduction

*Synechococcus elongatus* PCC7942 is considered a model organism since the early 1970s, when successful transformations of exogenous DNA were performed for the first time in a cyanobacterium [1]. In particular, it has been used as a paradigm for the study of circadian rhythms in prokaryotes as it has been demonstrated that prokaryotes are capable of measuring time [2]. *S. elongatus* has a rod-shaped appearance, is oligotrophic having the ability to survive in freshwater environments with low nutrients and is considered an obligate autotroph [3,4]. The genus *Synechococcus* is among the most important photosynthetic bacteria in the marine environment as it accounts, after different estimates, for about 25% of the primary production in marine habitats [5]. Like all cyanobacteria, *Synechococcus elongatus* uses CO_2 _as carbon source and light as energy source, which explains the interest in exploring its potential as a photo-biological cell factory for the production of valuable compounds for various applications. Potential applications are broad in this sense and research has been focused in the production of diverse metabolites of industrial interest, such as different types of biofuels [6] like hydrogen [7], among others. Its role as a model organism and the unique properties of this photosynthetic prokaryote illustrate why *Synechococcus elongatus* PCC7942 constitutes an interesting target for metabolic engineering and the benefit of developing, for the first time, a genome-scale metabolic model of this bacterium.

The development of genome sequencing and genetic mapping together with omics-science paved the way towards the quantitative study of biological systems. Thus, systems biology has emerged as a promising predictive science on a large and quantitatively deep scale [8], aiming at engineering of metabolic pathways and their capabilities [9]. Biological systems dynamics are inherently nonlinear and show functional synergies that may lead to emerging properties [10]. The construction of metabolic networks is, not only a compilation of chemical reactions, but also a gathering of exchange ratios, metabolic fluxes, and other type of biological constraints that make possible the *in silico* analyses of the organism’s behaviour. These analyses have been used by researchers to design metabolic engineering strategies in a variety of problems [11,12,13,14].

The genome-scale metabolic network reconstruction is based on genetic information available on the organism of interest. The *Synechococcus elongatus* PCC7942 genome was sequenced, annotated and published in 1980 [15,16,17]. In order to build a meaningful model, experimental data are required together with established knowledge such as physiological and biochemical information accessible from the literature, journal articles, experiments and databases,. In certain cases, lack of clarity and quality in published data, such as mistakes in entries, false negatives and false positives undermines the quality of the reconstructed models making their simulations worthless [18]. The relationships between complex metabolic processes usually falls to properly determine the processing of substrates into products and their stoichiometry, if a transformation is spontaneous or catalyzed by enzymes or if cofactors are involved. Moreover, the subcellular localization of the reactions and some thermodynamic aspects such as irreversibility must be known [19].

The usefulness of genome-scale metabolic models has been demonstrated through several computational analyses. Constraint-based approaches, such as flux balance analysis (FBA), are among the most common ones used to simulate phenotypic behavior under imposed physiological and/or genetic conditions [20,21,22]. FBA aims to obtain, through the optimization of a cellular objective (usually growth), the space of allowable flux distributions of a biological system under steady-state conditions. The optimization problem is subject to a set of constraints associated with lower and upper bounds in every reaction, which are defined by thermodynamic and experimental data. Finally, the resulting flux distribution can be contrasted with* in vivo* information and, thus, the metabolic model can be used for further analyses [23,24].

Presently, the process of reconstruction is long and arduous mainly due to its manual construction and proper quality-control check [25]. Some efforts have been directed to automate the metabolic reconstruction, or at least some parts of it, in order to cut down the time needed for such an endeavor. However, these efforts have been hampered due to problems in database information consistency and genome annotations [26]. Thereby, resulting algorithms are still unable to generate quality metabolic networks models as a basis for predictive analysis [25]. Several protocols have been published to define in detail each one of the steps of a proper reconstruction, as well as the software packages and databases that can assist in this labor [19,25,26,27]. For instance, Thiele and Palsson described in a very instructive way the process of debugging and validation [24].

Here, we present the manually curated metabolic reconstruction for *Synechococcus elongatus* PCC7942. The current model, *i*Syf715 features a detailed biomass equation including all the building blocks that are needed for a flux distribution simulation. Moreover, FBA analysis is performed to assess the accuracy of the model and to explore possible biotechnological production strategies.

With today’s energy shortage concerns, having seemingly infinite energy source of light represents an interesting avenue for research and development. Therefore, *i*Syf715 is a step towards the development of photo-biological production platforms for the synthesis of several compounds of industrial interest.

## 2. Results and Discussion

In the reconstruction of a genome-scale metabolic model the reliability of the model depends critically on the quality of the data used. Nowadays, several databases (Supplementary File S1) can be used to obtain reliable metabolic reactions and annotated genome sequences for the organism of interest. The reconstruction of the model of *Synechococcus elongatus* PCC7942, termed *i*Syf715 in the present work, started with an exhaustive data search and gathering of genome sequences and annotation files from the NCBI Entrez Gene database [28]. The rationale behind the name is that the “*i*” of the name refers to an *in silico* model, followed by the organism database identifier (e.g., KEGG ID) and then the number of genes whose information is included in the model.

**Figure 1:**
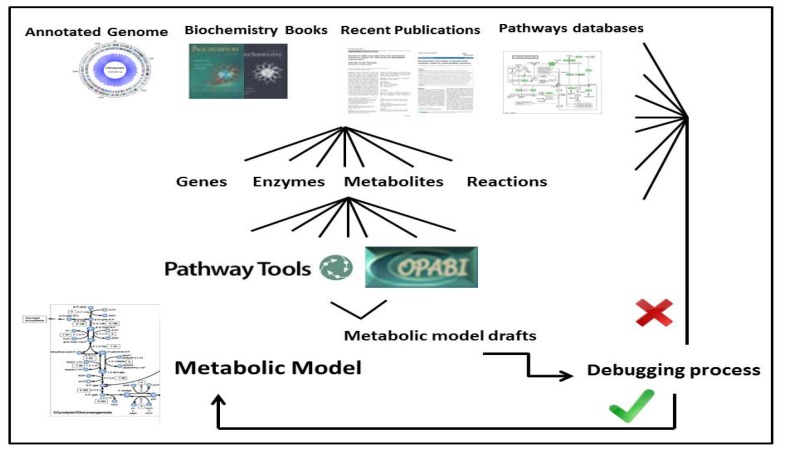
Genome-scale metabolic reconstruction process.

The files, of which descriptions can be found in Table 1, were used as input for the software Pathway Tools [29] in order to build a database with all the genes, proteins and metabolites present in the cyanobacteria. The list of reactions and cognate genes was then retrieved with this software. Alternatively, we used the COPABI computational platform to build a similar database and to automatically generate the metabolic model following probabilistic criteria of uniqueness and completeness [30]. The algorithm allows identifying and filling gaps in a given pathway, choosing a completeness value comparing the available information of our specific metabolic reactions (e.g., reactions related with buildings blocks) to a general pathway (meaning, ideally, “all” metabolic reactions in nature, or the meta-metabolism). This completeness value is a probability that the missing reaction can occur in our metabolic network. Furthermore, this platform gives the possibility to exclude duplicated reactions, allowing the inclusion and correction of many isoenzymes, through the uniqueness value. This strategy was conceived in order to double check the results of both software. Figure 1 summarizes the whole process.

**Table 1:** Summary of genome features of *Synechococcus elongatus* PCC7942.

	Chromosome	Plasmid pANL	Plasmid pANS
**Length of DNA (base pairs)**	2,695,903	46,366	7835
**G+C (%)**	~55.47	52.9	~59
**RNA genes**	54	-	-
**rRNA genes**	6	-	-
**tRNA genes**	45	-	-
**Other RNA genes**	3	-	-
**Protein genes**	2856	50	8
**With predicted function**	1682	17	-
**Without predicted function**	1174	33	-
**Total genes**	2906	50	8

The first draft included 672 genes coding for 540 enzymes that participated in 898 reactions. At this point we thoroughly checked the model identifying reactions that had no corresponding enzyme-coding gene assigned, but that needed to be included in the model, as well as removing reactions related to genetic replication, gene expression and cell division that were not meant to be included in the model [19]. Characteristics of this first draft and the final model version can be studied in Table 2. We verified EC numbers and stoichiometry of the reactions with several databases, such as KEGG pathway [31] and MetaCyc [32], as well as a complete literature examination from different biochemistry books [33,34,35,36]. If no conclusive outcome came from these sources, certain published metabolic models, such as *Synechocystis* sp. PCC6803 [11,12], cyanobacteria of the same phylogenetic Phylum as *Synechococcus* *elongatus* PCC7942 served as a reference to solve these issues.

**Table 2:** Distribution of elements in the database retrieved from Pathways Tools and the final model, *i*Syf715.

General Overview	*i*Syf715
Genes	715
Metabolic reactions	851
Metabolites	838
Enzymes	530
Multimeric enzymes and enzymatic complexes	79
**Reactions overview**	
Reversible reactions	326
Irreversible reactions	525
**Reactions with assigned genes**	**735**
Enzymatic conversion	710
Protein-mediated transport (active and passive-mediated transports)	25
**Reactions with no cognate genes**	**116**
Non-enzymatic conversion (spontaneous)	13
Passive transport reactions (simple diffusion)	16
EC reactions not annotated	76
Unassigned reactions	11

In order to render chemical conversions coherent, all elements were balanced. Protons have been balanced, by accounting the total number of each chemicals element on both sides of the associated-reactions in model. Electrons were balanced in the chemical reactions, even though we are unable to know the reducing state of metabolites in many reactions. We considered the principle of conservation of reducing power and have corrected the REDOX reactions based on an approximate balance of *electron-donor* and *electron*-acceptor. As some of the reactions included in these databases are usually reported in a non-specific form (e.g., an electron acceptor or an alcohol), corresponding organism-specific metabolites had to be identified [25]. In addition, reactions catalyzed by multimeric enzymes or enzymatic complexes were described as a single reaction [19]. The BRENDA database [37] helped to identify 325 reactions that were found to be reversible in the model. If no conclusive evidence was reported, reactions were set to be reversible.

Through these analyses, we assessed the possibility of including missing cofactors (e.g., water molecule or hydrogen ion, among others) in some reactions, like the ones catalyzed by hydrolases, oxidoreductases or transferases. If state of the art was unable to specify a single cofactor requirement, like NADH or NADPH, two reactions were included in the metabolic network.

No lumped reactions were left in the model to enable the tracing of the reactions’ fluxes. Several reactions were found to be necessary for the synthesis of monomers, precursors or building blocks, but had no corresponding enzyme-coding gene assigned. We included these reactions to allow the formation of biomass, which was an objective function of the simulations. Whenever such a reaction was included in the model, it was mentioned explicitly.

In the biomass reaction, proteins, nucleic acids, lipids, carbohydrates, and other essential organic compounds, are drained together in a virtual reaction that evolves a mole of biomass. The ratios of each one of these precursors, ideally determined experimentally, are added as stoichiometries in the reaction.

Additionally, some transport systems across the membrane such as: phosphate, water, sulphate, nitrate, ammonia, as well as carbon monoxide and hydrogen peroxide transport, were included in the model and properly bounded. Some of the reversible reactions involving NADH and NADPH were constrained to be irreversible so that spurious transhydrogenation was controlled.

Another essential point in the debugging process was the removing of internal loops that are thermodynamically infeasible, for instance futile cycles, like substrate cycles described in [33] and Type III-extreme pathway [38]. Blocking these reactions is crucial since several constraint-based approaches, such as Flux Balance Analysis [20], do not account for regulation, thus, futile cycles cannot be shut down otherwise and simulations could retrieve unnatural flux behaviors.

The resulting network of this reconstruction process encompasses all known metabolites that take place in *Synechococcus elongatus* PCC7942 and consists of 851 metabolic reactions and 838 metabolites (see Table 2). The bulk of reactions are catalyzed by 530 enzymes encoded by 715 genes. The presence of protein complexes and multimeric enzymes, explains the differences between the number of enzymes and genes. Additionally, a set of reactions with no cognate genes is present in *i*Syf715: 13 non-enzymatic (spontaneous) conversions, 16 simple diffusion reactions, and 11 unassigned reactions (the majority according to the KEGG report). During the reconstruction process, 54 external metabolites and 40 exchange reactions were included. In short, a total of 76 reactions not annotated in the genome were included in the model on the basis of biochemical evidence or physiological considerations. Examples of these are the genes that encode for malate synthase (EC 2.3.3.9) and isocitratelyase (EC 4.1.3.1), whose enzymatic activities have been measured [39], but do not have a cognate ORF associated to them, and whose presence is necessary to complete the glyoxylate shunt.

The final model includes central metabolic pathways, such as the glycolysis/gluconeogenesis pathway, the Calvin-Benson cycle, the pentose phosphate pathway, incomplete reactions within the tricarboxylic acid cycle (TCA), as well as the complete set of anabolic pathways involved in the biosynthesis of chlorophyll, glycogen, amino acids, lipids, nucleotides, vitamins, cofactors,* etc.* Pathways for glyoxylate synthesis (via ribulose-1, 5-bisphosphate carboxylase/oxygenase and the shunt across TCA cycle), and amino sugars metabolism are also included.

Photosynthetic electron transfer associated with the thylakoid membrane is represented as a set of 10 separate reactions, including light captured by photosystem II (PSII) and photosystem I (PSI), electron transfer between the two photosystems, and cyclic electron transfer which involves PSI and ferredoxin.

Working model files can be obtained in Supplementary information. Additionally, *i*Syf715 model on SBML format was deposited in BioModels Database [40] and assigned the identifier MODEL1407310000.

### 2.1. Network Topology and Connectivity Analysis

The network topology analysis of *i*Syf715 can help in the understanding of how metabolites and their interactions determine their metabolic function in the cell. As many studies have shown, most of these networks are scale-free and thereby the nodes connection can be estimated by a power-law degree distribution [41,42,43,44]. Most connected metabolites distribution in the *i*Syf715 network and a comparison with their presence in other microbial genome-scale metabolic networks can be seen in Table 3.

**Table 3:** Most connected metabolites in *S. elongatus* PCC7942 and other genome-scale metabolic models.

Metabolic Hubs	Neighbors in *i*Syf715	Neighbors in *i*Syn811 [12]	Neighbors in *E. coli* [45]	Neighbors in Yeast [19]
H_2_O	243	219	697	-
Phosphate	169	112	81	113
ADP	159	111	253	131
ATP	148	136	338	166
H^+^	149	153	923	188
Diphosphate	110	84	28	-
CO_2_	69	72	53	66
AMP	74	21	86	48
NADPH	74	68	66	57
NADP^+^	72	68	39	61
L-glutamate	52	44	52	56
NAD^+^	46	52	79	58
NADH	45	48	75	52
oxygen O_2_	45	40	40	31
*S*-adenosyl-L-methionine	37	28	18	19
Ammonia	44	28	22	-
coenzyme A	29	23	71	39
Pyruvate	32	20	61	20
L-glutamine	30	21	18	23
Glutathione	32	26	17	10
S-adenosyl-L-homocysteine	25	24	12	14

A few metabolites are involved in many reactions and are widely used by diverse metabolic machineries; on the other hand, many metabolites have very few connections. These highly connected metabolites are often referred as metabolic hubs; these are the metabolites around which metabolism is organized in a bow tie manner [46]. Unsurprisingly, water is the well-connected compound. Its role both as a substrate or product in reactions, such as reduction-oxidation, hydrolysis and condensation, is well known. As we can see in Table 3, other most well connected metabolites include carrier molecules like: ADP, ATP, NADP^+^, NAD^+^, phosphate, and oxygen, which are related to energy transport, energy storage and redox pathways; a few amino acids, peptides and their precursors (L-glutamate, L-glutamine and glutathione); and key components in the porphyrin and chlorophyll metabolism (S-adenosyl-L-methionine/S-adenosyl-L-homocysteine). Additional well connected metabolites are ammonia, coenzyme A and pyruvate, which constitute either the substrates or products of many central metabolic pathways, like glycolysis, tricarboxylic acid (TCA) cycle, glyoxylate shunt, and amino acids metabolism.

**Figure 2:**
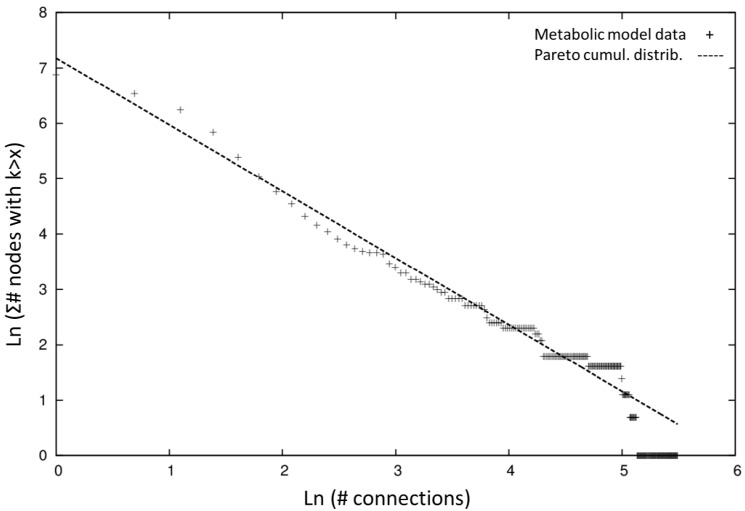
Connectivity distribution of the *i*Syf715 metabolic model using the cumulative distribution towards the right via Pareto cumulative distribution.

For the connectivity distribution analysis of *i*Syf715 we used a systematic mathematical approach: the Pareto’s law in terms of the cumulative distribution function (*P (K*>*k)~k^−γ + 1^*) to get a proper fit [47,48].We used the cumulative distribution rather than picturing a log-log scale plot of the distribution of connections counts among number of nodes, so that the distribution tail smoothed out in the cumulative distribution and no data were “obscured” as in the logarithmic binning procedure [49]. Our analysis of the data from Table 3 leads us to think the *i*Syf715 network is reasonably characterized by a power-law distribution (*γ* = 2.203) with high non-uniformity, as we can see at the cumulative distribution towards the right of Figure 2. The biological significance of this hierarchical connectivity is said to be related to an evolutionary process, where the hubs were the first compounds that were present in the earliest cells predecessor’s metabolism [50]. Metabolic hubs represent effective targets for metabolic engineering and should be considered in the design of strategies for the production of other metabolites.

### 2.2. Simulation of the Model

We performed a constraint-based flux simulation by using the Flux Balance Analysis (FBA) [20] algorithm. This approach has been used to simulate exponential growth in bacteria and assumes that the cells grow optimally using a given amount of carbon and energy source. Another assumption is the steady state of intracellular reactions in the exponential phase. As the number of equations (reactions) is much larger than the number of variables (metabolites), we have an underdetermined system of equations, which is solved by applying a steady state and optimizing for an objective function (cell growth or biomass evolution). This optimization was done with linear programming, which requires the establishment of boundary parameters for the uptake. The solution for this mathematical problem (see Methods) is a flux vector, where each reaction has a flux value. Model validation usually focuses on testing whether the growth capabilities or any particular objective flux corresponds to a given set of experimental flux data.

*Synechococcus elongatus* PCC7942 is an obligate photoautotroph organism, thus we defined a set of constraints for this growth conditions (detailed in Supplementary file S1, Table S2). A two-step optimization procedure was applied as in previous works [11]. The first step was the maximization of biomass growth while the light intake was unconstrained. Next, the maximum growth value was incorporated as a constraint to minimize the light uptake rate (the second step). This was designed with the aim to estimate physiologically meaningful photon uptake values that tallied experimental growth measurements. Biomass synthesis, a theoretical abstraction for cellular growth, is considered as a drain of some metabolite intermediates, into a general biomass component [11]. We looked upon information about weight fractions of macromolecules and monomers to reflect the composition of biomass. Frequently, data related to the relative amounts of these metabolites are not available in literature, or the published information is shown in a particular physiological condition not usable for our goals. As long as possible, the weight fractions were updated to reflect the specifics of *Synechococcus elongatus* PCC7942. The quantities measured in other phylogenetically related biological systems could be a close approximation to the metabolic reality of the concerned organism. In the present work we have used the biomass composition reported in Table 4. For details, please find a more detailed explanation of biomass equation in Methods and Supplementary File S1.

For the estimation of the theoretical maximum illumination we calculated the surface area per weight of biomass. We considered a spheroid geometry of the cell with a length of 3.57 ± 0.12 μm, a width of 1.47 ± 0.09 μm and a dry weight of 3.87 ± 0.03 ng [51] and we assumed an irradiance value of 0.156 mE m^−2^ s^−1^ in a 12:12 hours photoperiod as the value with the highest growth rate experimentally determined [51]. Taking into account these data the theoretical maximum illumination that would reach the cell membrane in our model was estimated as 1.96 mE gDW^−1^ h^−1^. The first optimization was carried out by constraining the CO_2_ and HCO_3_^−^ uptake rates at 1.99 mmol gDW^−1^ h^−1^ [52] and maximizing growth. Then we imposed that specific growth rate as a condition and we minimized the photon uptake rate. That is, finding the minimal quantity of photons needed for the metabolism to work with the experimentally determined carbon uptake. In this way, we estimated physiologically meaningful photon uptake values, which do tally with experimental growth measurements [11,53]. The specific growth rate for *i*Syf715 resulted to be 0.05987 h^−1^ with a photon uptake rate of 0.1 mE gDW^−1^ h^−1^ on each photosystem.

Due to the biphasic nature of cyanobacterium growth, we look for reported data for exponential growth phase with which to compare our simulation. The maximum specific growth rate of S*ynechococcus* *elongatus* PCC7942 has been reported from 0.0519 h^−1^ to 0.0551 h^−1 ^[52,54], despite scarce information about studies on optimization of the specific growth rate of this prokaryote. The slight difference of our simulated growth with the experimental data could be the result of several factors, including regulation, stress and feedback inhibition, which cannot be captured in constraint-based stoichiometric models. Moreover, the growth of many laboratory strains are not consistent with the computed optimal by FBA because they are not necessarily evolved for growth maximization [55,56]. These results are, therefore, an overall acceptable validation of the genome-scale metabolic model.

**Table 4:** Biomass composition of the *S. elongatus* PCC7942 metabolic model.

Metabolites	mmol/gDW	Metabolites	mmol/gDW
Proteins	0.000459	**Ribonucleotides**	
**Carbohydrates**		AMP	0.140389293
Glycogen	0.53439	UMP	0.140389293
**Antenna chromophores**		GMP	0.123745851
Zeaxanthin	0.00079	CMP	0.123745851
Beta-carotene	0.000875	**Lipids**	
Trans-lycopene	0.00820225	14C-lipid	0.028
Chlorophyll a	0.0057	16C-lipid	0.0042
Phycocyanobiline	0.0285	18C-lipid	0.00448
**Deoxyribonucleotides**		(9Z)16C-lipid	0.0066
dATP	0.0201156	(9Z)18C-lipid	0.00625
dTTP	0.0201156		
dGTP	0.02538445		
dCTP	0.02538445		

**Figure 3:**
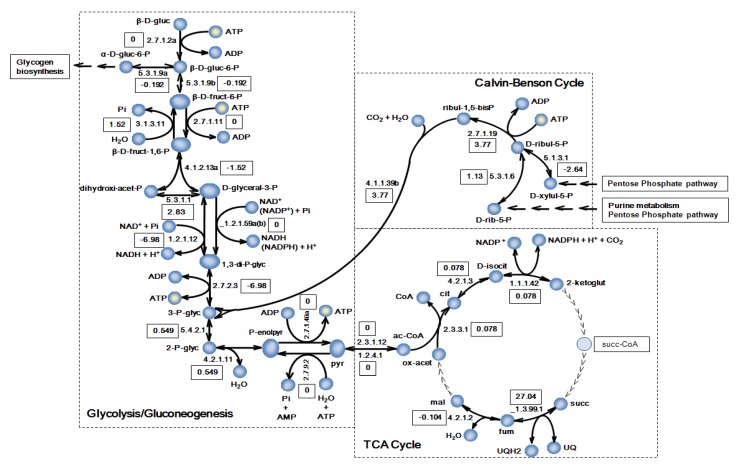
Flux landscape of the *i*Syf715 metabolic model. Abbreviations: β-D-gluc = β-D-glucose; β-D-gluc-6-P = β-D-glucose-6-phosphate; α-D-gluc-6-P = α-D-glucose-6-phosphate; β-D-fruct-6-P = beta-D-fructose-6-phosphate; β-D-fruct-1,6-P = β-D-fructose-1,6-bisphosphate; dihydroxi-acet-P = dihydroxy-acetone phosphate; D-glyceral-3-P = D-glyceraldehyde-3-phosphate; Pi = phosphate; 1,3-di-P-glyc = 1,3 diphosphateglycerate; 3-P-glyc = 3-phosphoglycerate; 2-P-glyc = 2-phosphoglycerate; P-enolpyr = phosphoenolpyruvate; pyr = pyruvate; ac-CoA = acetyl-CoA; ox-acet = oxaloacetate; mal = malate; fum = fumarate; UQH2 /UQ = reduced ubiquinone/oxidized ubiquinone; succ = succinate; succ-CoA = succinyl-CoA; 2-ketoglut = 2-ketoglutarate; D-isocit = D-isocitrate; cit = citrate; CoA = coenzyme A; ribul-1,5-bisP = D-ribulose-1,5-bisphosphate; D-ribul-5-P = D-ribulose-5-phosphate; D-rib-5-P = D-ribose-5-phosphate; D-xylul-5-P = D-xylulose-5-phosphate.

Furthermore, we studied split-flux reactions, nodes in the network where flux can go in two or more directions and that are very good checkpoints to see if complex metabolic behavior has been properly captured by the metabolic model (Figure 3 depicts the relevant reactions vector). The flux landscape of the model shows a flux distribution directed towards the CO_2 _fixation at the Calvin-Benson cycle, as expected of a photosynthetic cell that uses light and carbon substrates to build complex carbohydrate molecules. The solution space reveals that the autotrophic growth flows in the gluconeogenic direction (see Figure 3). Unsurprisingly, RuBisCO (reaction “4.1.1.39b”) and carbonic anhydrase (reaction “4.2.1.1b”) have high fluxes as they are entry points of carbon dioxide and carbonic acid, the carbon sources of the system. The demand of ATP and NADPH is supplemented by the photosynthetic processes. Depending on the needs of each of them, photosynthesis will be linear or include a cyclic part if more ATP is required. *i*Syf715 shows significant flows over the ferredoxin-NADP^+^ reductase (reaction “_1.18.1.2”) and across the ATPase (reaction “_3.6.3.14”), the governing complex in the photophosphorylation process. Thus, *i*Syf715 is flexible enough as to incorporate ATP/NADPH ratios, following the hypothesis that the existence of both electron transports must be essential for efficient photosynthesis [57].

The *i*Syf715 model displays the activation of the production of glycogen through the ADP-D-glucose synthesis, which serves as a form of carbon storage. The glyoxylate shunt is basically inactive, and the TCA cycle is incomplete. As known in many metabolic engineering works, acetyl-CoA stands as a possible metabolite of industrial relevance in *Synechococcus* as it can be the starting point of biosynthesis of several amino acids and fatty acids, which are of high importance to the production of biofuels [6]. In this sense, previous studies have worked on engineering reactions around acetyl-CoA, and have reported promising results in microorganisms genetically engineered to produce added-value metabolites [58,59,60].

It is noteworthy that reactions such as those catalyzed by glucokinase (reaction “2.7.1.2a”), phosphofructokinase (reaction “2.7.1.11”) and pyruvate kinase (reaction “2.7.1.40a”), associated with de novo synthesis of carbohydrates, exhibit no flux, which is in correspondence with photoautotrophic growth conditions [33,61]. Others, like those catalyzed by glucose-6-phosphate isomerase (reaction “5.3.1.9a,b”), fructose 1,6-bisphosphate phosphatase (reaction “3.1.3.11”) and phosphoribulokinase (reaction “2.7.1.19”), as well as those that produce D-ribulose-1,5-bisphosphate and catalyzed by ribose-5-phosphate isomerase (reaction “5.3.1.6”) and ribulose-phosphate 3-epimerase (reaction “5.1.3.1”), are active, as should be in photoautotrophic conditions. Finally, we would like to focus on the high flux value of the reversible reaction “_1.3.99.1” catalyzed by succinate dehydrogenase. In this case the direction implies the succinate oxidation to fumarate reducing ubiquinone. The synthesis of fumarate is an essential reaction since it represents an intermediate node in many metabolic pathways that yield building blocks for biomass formation; such as uridine monophosphate (UMP) and aspartate. It is known that this cyanobacterium does not have abundance of complex morphological characteristics [62], therefore, the reduced ubiquinone could be oxidized by other processes, such as photosynthesis electronic transfer, contributing to the formation of NADPH and ATP without triggering their classical synthesis reactions’ fluxes.

The genome-scale metabolic model of *Synechococcuselongatus*PCC7942, *i*Syf715, is available in Supplementary files S2, S3 and S4 and its results can be traced in Supplementary file S5.

## 3. Methods

### 3.1. Genome-Scale Metabolic Network Reconstruction

Pathway Tools [29] and COPABI [30] software were used to construct a specific database of genes, proteins, enzymes and metabolites. All the genome and annotation files of *Synechococcus elongatus* PCC7942 were downloaded from the NCBI Entrez Genome [28] repository as of 24 February, 2011. The network was retrieved by Pathway Tools and was checked using COPABI filling gaps algorithms and different databases, like KEGG pathway database [31], MetaCyc [32], and BRENDA Enzyme database [37]. See Supplementary File S1 for a complete list.

*i*Syf715 model on SBML format was deposited in BioModels Database [40] and assigned the identifier MODEL1407310000.

### 3.2. Linear Programming for Flux Balance Analysis

All biochemical reactions in iSyf715 were formulated as a stoichiometric model, such as:

*S*·*v*

Here, *S* is a stoichiometric matrix that contains the stoichiometric coefficients from all internal (balanced) metabolites, whereas *v* is a flux vector that corresponds to the columns of *S*. Using an approach known as flux balance analysis or FBA [20], the former equation can be solved using linear programming and a given set of experimentally-driven constraints. In FBA the number of reactions is often larger than the number of metabolites, making the system underdetermined. Generally, in order to solve the problem and obtain a feasible solution for the intracellular fluxes, a steady state and an optimization criterion on metabolic balances has to be imposed. Steady state can be imposed so that:

*S*·*v* = 0

In addition, the optimization criterion can be set by maximizing one biochemical reaction, such as the biomass evolving equation. Additionally, boundary conditions have to be used to be able to simulate the steady state.

For instance,

Max (*v_j_*)   subject to *S* · *v_j_*_ =_ 0   ∀*j*∈ N

*v_j, irr_*∈ R^+^

*v_j, rev_*∈ R

*v_j, const_*∈ R, *v_min_*<*v_j,const_*<*v_max_*

*v_j,uptake_*∈ R, *v_min_*<*v_j,uptake_*<*v_max_*

Where *v_j_* is the rate of the *j^th^* reaction. The elements of the flux vector *v* were constrained for the definition of reversible and irreversible reactions, *v_j_*, _rev_ and *v_j, irr_*, respectively. Additionally, two set of equations were established, *v_j, const_*, constraining metabolic reactions, and *v_j, uptake_*, uptake reactions, which were bound through experimentally determined values from the literature. Biomass synthesis was considered as a drain of precursors or building blocks into hypothetical biomass component. Flux through biomass synthesis reaction, being the biomass formation rate, is directly related to growth of the modeled organism. Simulations were performed using the OptGene software [63], later termed BioOpt [64,65]

More information about the modeling mechanics can be found in Montagud* et al*., 2010 [11]. Readers will find in Supplementary file S2 the model file where all reactions and simulated constraints are depicted and ready to be used in OptGene software [63].

### 3.3. Biomass Composition

Biomass synthesis was considered as a drain of precursors or building blocks into a hypothetical biomass component. Precursors considered were amino acid, carbohydrates, chromophores, nucleic acids, and lipids. All data were retrieved from published information as described in Supplementary File S1. Flux through the biomass synthesis reaction, being the biomass formation rate, is directly related to growth of the modeled organism [20]. Table 4 shows the biomass composition that was considered in the current metabolic model and a detailed description can be found in Supplementary File S1.

## 4. Conclusions

We have successfully reconstructed the first genome-scale metabolic network for *Synechococcus elongatus* PCC7942, called *i*Syf715. The curated model represents an up-to-date database that encompasses all knowledge available in public databases, scientific publications and textbooks on the metabolism of this cyanobacterium. The model has been compiled in OptGene and SBML to enable its use with different software.

Our model includes 851 metabolic reactions and 838 metabolites, as well as the information from 715 genes. Moreover, we identified 76 enzymatic reactions needed for the correct function of the metabolism, but with no annotated cognate gene. These genes are interesting targets for experimental studies as we have seen that their presence is required in order to build up the basic cellular components.

From the topological perspective the characteristics of the model are very similar to other published organisms’ providing support for an evolutionary study of the structure and organizational properties of metabolic networks, in the line of recent works [66]. The connectivity analysis of the network model using the Pareto cumulative distribution shows scale-free behavior with a high non-uniformity and a hierarchical connectivity of the metabolites, which is typical of biological networks and points towards functional properties discussed in other works [46].

Flux balance analysis of the model was applied in order to simulate the autotrophic growth rate of the cyanobacteria. The *i*Syf715 was able to simulate growth, which approached experimental values as well as characteristic reactions directions and split fluxes specific of this cyanobacterium. Moreover, typical flux characteristics of photosynthetic cells, such as active Calvin-Benson cycle or production of glycogen through the ADP-D-glucose synthesis were observed in our simulations.

In conclusion, the genome-scale metabolic network of *Synechococcus elongatus* PCC7942 (*i*Syf715) will be a valuable tool for industrial applications and fundamental research. Its use will allow the study and experimentation of this cyanobacterium as a possible light-driven production chassis for metabolites of industrial interest, in the line of *Synechocystis* sp. PCC6803 [11,12], *Cyanothece* sp. ATCC 51142 [67] or Synechococcus sp. PCC 7002 [68].

## Supplementary Information

Supplementary materials can be accessed at: http://www.mdpi.com/2218-1989/4/3/680/s1.

- Supplementary File S1: Table S1, Table S2 and detailed description of biomass reaction.
- Supplementary File S2: iSyf715 model with constraints ready to be simulated in OptGene/BioOpt.
- Supplementary File S3: iSyf715 model with gene/reaction association in Excel.
- Supplementary File S4: iSyf715 model in SBML.
- Supplementary File S5: iSyf715 metabolic flux values.

## Supplementary Files

Supplementary File 1
