# Supplementary material for: Generation and Evaluation of a Genome-Scale Metabolic Network Model of Synechococcus elongatus PCC7942
Source: Metabolites. 2014 Aug 20;4(3):680–98. doi: 10.3390/metabo4030680 (PMC4192687; doi:10.3390/metabo4030680)
Supplement: Supplementary File 1 [file metabolites-04-00680-s001.zip › metabolites-04-00680-supplementary/Supplementary File S1.pdf]

Supplementary File S1

Table S1. Examples of databases that can assist during the reconstruction process of a genome-scale metabolic network.

**Genomes and genetic information**	
**NCBI Entrez Gene (National Center of Biotechnology Information)**	http://www.ncbi.nlm.nih.gov/sites/entrez
DDBJ(DNA Data Bank of Japan)	www.ddbj.nig.ac.jp
EMBL-Bank (*Europe's nucleotide sequence database*)	*www.ebi.ac.uk/embl*
KEEG (Kyoto Encyclopedia of Genes and Genome)	*http://www.kegg.com*
**BioCyc (collection of genomes and metabolic pathways)**	http://www.biocyc.org
**Ensembl (provides annotations of some vertebrates and eukaryotic genomes)**	http://www.ensembl.org
**JGI Genomes (DOE-Joint Genome Institute, is a repository of various genomes)**	http://genome.jgi.doe.gov
**Ecocyc (*Escherichia coli* K-12 MG1655 database)**	http://www.ecocyc.org
**MGI (Mouse Genome Informatics)**	http://www.informatics.jax.org
**MaizeGDB (Maize Genetics and Genomics Database)**	http://www.maizegdb.org
**SGD (*Saccharomyces* Genome Database)**	http://www.yeastgenome.org
**Wormbase (Database which include the genome of *Caenorhabditis elegans*)**	http://www.wormbase.org
**ZFIN (Zebra Fish Information Network)**	http://zfin.org
**FlyBase (A Database of Drosophila Genes & Genomes)**	http://flybase.org
**Tair (The *Arabidopsis* Information Resource)**	http://www.arabidopsis.org
**CyanoBase (Database of cyanobacteria genomes)**	http://genome.kazusa.or.jp/cyanobase
**Metabolic pathways**	
KEEG (Kyoto Encyclopedia of Genes and Genome)	*http://www.kegg.com*
**BioCyc (collection of genomes and metabolic pathways)**	http://www.biocyc.org
**MANET database (Molecular Ancestry Network)**	http://www.manet.uiuc.edu

**Reactome (Pathways annotations database)**	http://www.reactome.org
**BioCarta(Biological Pathways databases)**	http://www.biocarta.com
**Enzymes**	
**ExPASy-Enzyme(Enzyme nomenclature database)**	http://www.expasy.org/enzyme
**BRENDA (The Comprehensive Enzyme Information System)**	http://www.brenda-enzymes.org
**IntEnz (Integrated relational Enzyme database)**	http://www.ebi.ac.uk/intenz
**SABIO (Biochemical Reaction Kinetics Database)**	http://sabiork.h-its.org
**CAZy (Carbohydrate Active enzyme database)**	http://www.cazy.org
**Metabolic compounds**	
**EBI-ChEBI (Chemical Entities of Biological Interest)**	http://www.ebi.ac.uk/chebi
**PDB (The Chemical Component Dictionary)**	http://remediation.wwpdb.org/ccd.html
**LIPIDMAPS (LIPID Metabolites and Pathways)**	http://www.lipidmaps.org
**LipidBank (Database of the Japanese Conference on the Biochemistry of Lipids (JCBL))**	http://lipidbank.jp
**KNApSAcK (A Comprehensive Species-Metabolite Relationship Database)**	http://kanaya.naist.jp/KNApSAcK

Table S2. Principal constraints across the autotrophic growth condition. Units in mmol $gDW^{-1} h^{-1}$.

Constraints	Values in first Optimization*	Values in second Optimization*
Light input in PSI	0; 1.96	0; 0.1
Light input in PSII	0; 1.96	0; 0.1
CO_2 uptake rates	0; 1.99	0; 1.99
HCO_3^- uptake rates	0; 1.99	0; 1.99
Nitrate uptake rates	160; 160	160; 160
CO uptake rates	-10; 10	-10; 10
Sulphate uptake rates	-104; 104	-104; 104

* Values indicate, consecutively, minimum and maximum boundaries.

Detailed explanation of biomass equation

The biomass growth is the most common objective function used to simulate the metabolic flux distribution, and it has become a standard to assess the flux analysis in metabolic engineering strategies [1, 2].

The formulation of biomass composition lies on the stoichiometric coefficients of all of the substances that are the molecular basis of the construction of a cell, their building blocks. They consist of linked monomeric units that make up the lowest level of structural hierarchy of the cell. In particular, biomass growth is expressed by transforming the building blocks, such as: amino acids, desoxyribonucleotides, ribonucleotides, lipids, carbohydrates, antenna chromophores, some cofactors, etc, into one mole of biomass. Thus, growth flux is defined as a metabolic flux utilizing these biosynthetic precursors, *Xm*, in the appropriate ratios to produce biomass:

$$\sum_{\text{all m}} d_m \cdot X_m \rightarrow \text{biomass}$$

where *dm* stands for the stoichiometric coefficients (or biomass fraction) of the metabolite *Xm*.

Finding information about weight fractions of macromolecules and monomers to reflect the composition of any organism is critical.

As a part of the reconstruction process we detailed a biomass equation for *S. elongatus* PCC7942. Little is known about the specific molecular quantities of this cyanobacterium. However, the previous study of Rosales-Loaiza *et al.* in *Synechococcus* sp., isolated from a hypersaline waterhole, served as a reference in the composition of total protein, chlorophyll a, β-carotene and zeaxanthin [3]. Nevertheless, the total protein quantity per gram of dry cell weight (gDW) is not enough to describe the composition of this macromolecule in the cell, as its monomeric composition is very diverse. Hence, we adapted the amino acid quantities by selecting the well-studied protein composition of *Synechococcus* sp. PCC 7002 metabolic model as a template [4], also in Table S3 and can be traced in reaction "_a protein" in Supplementary file S2. Anyway, by sensitivity analysis a work found that the optimal growth rates do not change drastically by varying the monomeric composition of the major macromolecules [5]. Because the photosynthetic carbon assimilation in cyanobacteria results in the accumulation of polysaccharides, mostly in the form glycogen according to [6], we defined the composition of total carbohydrates as the amount of this polymer. Here, we assumed the carbohydrate composition measured in *Synechococcus* sp. PCC 7002 [7]. We included values of carotenoid pigment, in this case trans-lycopene, as biomass precursors using data reported in *Synechosystis sp.* PCC 6803 [8]. Moreover, we estimated ratios between the concentrations of chlorophyll a and phycocyanobiline measured in *S. elongatus* [9]. Thus, phycocyanobiline´s amounts were incorporated into biomass equation according to the chlorophylls quantities fixed.

In addition, lipids coefficient were based on the data for *S. elongatus* PCC7942 [10]. Finally, the molar quantities for the desoxyribonucleotides and ribonucleotides were defined from the information available from the works of Herdman *et al.* and Allen and Smith, respectively [11, 12].

Table S3: Amino acid composition of *Synechococcus* sp. PCC 7002 [4].

**Amino acid counts of the proteome**	
Alanine	897
Arginine	526
Aspartate	518
Asparagine	374
Cysteine	102
Glutamine	576
Glutamate	614
Glycine	702
Histidine	197
Isoleucine	628
Leucine	128
Lysine	417
Methionine	194
Phenylalanine	406
Proline	512
Serine	548
Threonine	580
Tryptophan	149
Tyrosine	294
Valine	638

In order to take into account the energy cost of all reaction not considered by our metabolic model information on the maintenance energy requirements had to be included. This energy accounts for both growth associated and non-growth associated maintenance functions [13, 14]. Some of them are cells active transports, membrane potentials, turn-over of macromolecules, maintenance of concentration gradients (pH or osmotic pressure), mobility and the ATP cost required for the polymerization of amino acids and nucleotides. Being unable to find *Synechococcus* data, we used the same

maintenance energy requirements as *Synechocystis* sp. PCC6803 metabolic model [15,16].

References

1. Edwards, J.S.; Ibarra, R.U.; Palsson, B.Ø. *In silico* predictions of *Escherichia coli* metabolic capabilities are consistent with experimental data. *Nat. Biotechnol.* **2001**, *19*, 125–130.

2. Ibarra, R.U.; Edwards, J.S.; Palsson, B.Ø. *Escherichia coli* K-12 undergoes adaptive evolution to achieve *in silico* predicted optimal growth. *Nature.* **2002**, *420*, 186–189.
3. Rosales-Loaiza, N.; Guevara, M.; Lodeiros, C.; Morales, E. Crecimiento y producción de metabolitos de la cianobacteria marina *Synechococcus* sp. (Chroococcales) en función de la irradiancia. *Rev. Biol. Trop.* **2008**, *56*, 421–429.
4. Hamilton, J.J.; Reed, J.L. Identification of functional differences in metabolic networks using comparative genomics and constraint-based models. *PLoS ONE* **2012**, *7(4)*, e34670.
5. Varma, A.; Palsson, B.Ø. Metabolic Flux Balancing: Basic concepts, scientific and practical use. *Nature.* **1994**, *12*, 994–998.
6. Nakamura, Y.J.; Takahashi, A.; Sakurai, Y.; et al. Some cyanobacteria synthesize semi-amylopectin type α-polyglucans instead of glycogen. *Plant Cell Physiol.* **2005**, *46*, 539–545.
7. Xu, Y.; Guerra, L.T.; Li, Z., Ludwig, M.; et al. Altered carbohydrate metabolism in glycogen synthase mutants of *Synechococcus* sp. strain PCC 7002: Cell factories for soluble sugars. *Metab. Eng*. **2012**, *16*, 56–67.
8. Miao, X.; Wu, Q.; Wu, G.; Zhao, N. Changes in photosynthesis and pigmentation in an agp deletion mutant of the cyanobacterium *Synechocystis* sp. *Biotechnol. Lett.* **2003**, *25*, 391–396.
9. González-Barreiro, O.; Rioboo C.; Cid A.; Herrero C. Atrazine-induced chlorosis in *Synechococcus elongatus* cells. *Arch. Environ. Contam. Toxicol.* **2004**, *46(3)*, 301–307.
10. Martin-Creuzburg, D.; Sperfeld, E.; Wacker, A. Colimitation of a freshwater herbivore by sterols and polyunsaturated fatty acids. *Proc. R. Soc. B. rspb.* **2009**, *276(1663)*, 1805–1814.
11. Herdman, M.; Janvier, M.; Waterbury, J.B.; et al. Deoxyribonucleic Acid Base Composition of Cyanobacteria. *J. Gen. Microbiol.* **1979**, *111*, 63–71.
12. Allen, M.M.; Smith, A.J. Nitrogen chlorosis in blue-green algae. *Arch. of microbial.* **1969**, *69*, 114–120.

13. Stouthamer, A.H. The search for correlation between theoretical and experimental growth yields. *Microb. Biochem.* **1979**, *21*, 1–48.
14. Förster, J.; Famili, I.; Fu, P.; et al. Genome-scale reconstruction of the *Saccharomyces cerevisiae* metabolic network. *Genome Res.* **2003**, *13*, 244–253.
15. Montagud, A.; Navarro, E.; Fernández de Córdoba, P.; et al. Reconstruction and analysis of genome-scale metabolic model of a photosynthetic bacterium. *BMC Syst. Biol.* **2010**, *4*, 156–172.
16. Montagud, A.; Zelezniak, A.; Navarro, E.; et al. Flux coupling and transcriptional regulation within the metabolic network of the photosynthetic bacterium *Synechocystis*sp. PCC6803. *Biotechnol. J.* **2011,** *6*, 330–342.
